# An Epidemic and a Play at Manchester

**Published:** 1914-01-03

**Authors:** 


### An Epidemic and a Play at Manchester.

How little the outside world knows of the strenuous efforts put forth by the staff attached to our voluntary hospitals in order that Christmas may be a time of happiness and enjoyment to those placed under their care at this season! From early in December until Christmas has passed they are preparing and scheming for the enjoyment of others. The matron, sisters, and nurses of Ancoats Hospital, Manchester, must be congratulated on their endeavours to make Christmas Day a really happy one for the patients. The wards were all tastefully decorated. Sister Sykes, in her accident wards, had arranged long chains of laurels from the four corners of her wards, joining them to the chimney, which in these wards is situated in the centre. Over the opal shades of the electric light was fancy coloured paper with gilt edging, but so placed that the edge did not project beyond the opal shade, thus minimising the risk of the paper catching fire in the event of a bulb bursting. The men's wards, situated on the top floor, looked very pretty after Sister Cooper had finished her decorations. With an artistic eye this sister had placed round the walls of her medical and surgical wards chains of dark laurel leaves, while from each wire connecting the electric light to the ceiling she had attached about half-way down chains of leaves of a lighter hue, which, against the darker shade of the laurel leaves, showed up wonderfully well, and made the wards look bright and cheerful. On the tables vases containing beautiful pink lilies were placed, and the doorway connecting the two wards one might have taken for a bower, thus making the whole as pleasing a picture as one could wish to see. The women's wards, under the care of Sister Cadmore, looked very pretty in pink and gilt, and it was in these wards that the Christmas concert was given. A stage had been erected at the end of the medical ward, which was prettily draped with red, white, and blue cloth, adorned with gilt facings. The timber for this stage was kindly lent by Messrs. J. Southern and Sons, of Manchester, and it was erected by the hospital engineer, while the drapery was a gift from the chairman, Mr. W. Armitage. We are exceedingly sorry for Sister Bell, who mothers the wee bairns in the children's wards. For weeks past this sister and her staff of nurses have been busily engaged making Christmas stockings, obtaining toys and preparing for the Christmas-tree, but a damper was put on all their efforts by an outbreak of scarlet fever, with the consequent removal of one of the nurses and a little patient to the isolation hospital on Christmas Eve; and a further outbreak occurring on Boxing Day in the same ward all hopes of having a Christmas-tree entertainment were put to an end for the present.

On Christmas morning the nurses went carol singing round the wards, and following the usual custom presents of useful articles of clothing were given to the adult patients, and dolls, toys, etc., were given to the children. The house surgeon, Dr. Whitehead, dressed as a curate, the house physician, Dr. Bedale, as a clown, and the assistant house surgeon, Dr. F. Battison Smith, as an old-fashioned country farmer, kept the patients much amused all the afternoon. With red whiskers and beard, a short clay pipe, and a long white smock, Dr. Smith carried out his part excellently, the patients laughing heartily over his various antics. A good programme was presented to the patients at the concert given at six o'clock. Mr. W. R. Douglas, one of the honorary surgeons, and Mrs. Douglas were present and took part. The songs, beautifully rendered by Mrs. Douglas, were very much appreciated, as expressed by the many encores that were given.

The play entitled "Election Petition," by Mr. J. Cargill, was given by the nurses, through the kind permission of Messrs. French, of London. Sister White is to be congratulated on the result of her endeavours to make the play a success. The trouble and patience taken by her were amply rewarded by the excellent manner in which the nurses did their respective parts. The scene was a Court of Justice, and the time the year 1950, representing the final attainment of the women's suffrage movement, when the women shall rule and no man be allowed into Parliament. Nurse Chappell as Mrs. Justice Broadbrief, Nurse Smethurst as Miss Letitia Phadd, and Nurse McMahon as Miss Winifred Winsome, M.P., with the counsel for the petitioner and defendant, and the witnesses made a very pretty stage effect, and all those who took part must rejoice in their maiden efforts in amateur theatricals. After the play further songs were rendered by Mrs. Douglas and Nurse Smethurst, Mr. Mackie giving the "Rag-time Parson" and Dr. Bedale "Dum Dum," the encores received in each case being exceedingly well merited. Christmas Day ended with all lights out at eight o'clock.

